# TGF-β_1_ Potentiates the Cytotoxicity of Cadmium by Induction of a Metal Transporter, ZIP8, Mediated by the ALK5-Smad2/3 and ALK5-Smad3-p38 MAPK Signal Pathways in Cultured Vascular Endothelial Cells

**DOI:** 10.3390/ijms23010448

**Published:** 2021-12-31

**Authors:** Keisuke Ito, Tomoya Fujie, Masahiro Shimomura, Tsuyoshi Nakano, Chika Yamamoto, Toshiyuki Kaji

**Affiliations:** 1 Faculty of Pharmaceutical Sciences, Tokyo University of Science, 2641 Yamazaki, Noda 278-8510, Japan; 3A18702@ed.tus.ac.jp (K.I.); j3B13645@ed.tus.ac.jp (M.S.); t-nakano@rs.tus.ac.jp (T.N.); 2 Faculty of Pharmaceutical Sciences, Toho University, 2-2-1 Miyama, Funabashi 274-8510, Japan; t-fujie@phar.toho-u.ac.jp

**Keywords:** cadmium, endothelial cell, Zrt- and Irt-like protein transporter, transforming growth factor-β_1_, Smad2/3, p38 MAPK

## Abstract

Vascular endothelial cells cover the luminal surface of blood vessels in a monolayer and play a role in the regulation of vascular functions, such as the blood coagulation-fibrinolytic system. When the monolayer is severely or repeatedly injured, platelets aggregate at the damaged site and release transforming growth factor (TGF)-β_1_ in large quantities from their α-granules. Cadmium is a heavy metal that is toxic to various organs, including the kidneys, bones, liver, and blood vessels. Our previous study showed that the expression level of Zrt/Irt-related protein 8 (ZIP8), a metal transporter that transports cadmium from the extracellular fluid into the cytosol, is a crucial factor in determining the sensitivity of vascular endothelial cells to cadmium cytotoxicity. In the present study, TGF-β_1_ was discovered to potentiate cadmium-induced cytotoxicity by increasing the intracellular accumulation of cadmium in cells. Additionally, TGF-β_1_ induced the expression of ZIP8 via the activin receptor-like kinase 5-Smad2/3 signaling pathways; Smad3-mediated induction of ZIP8 was associated with or without p38 mitogen-activated protein kinase (MAPK). These results suggest that the cytotoxicity of cadmium to vascular endothelial cells increases when damaged endothelial monolayers that are highly exposed to TGF-β_1_ are repaired.

## 1. Introduction

Vascular endothelial cells cover the luminal surface of blood vessels with a monolayer and are in direct contact with the blood. Vascular endothelial cells control vascular tone and blood coagulation-fibrinolytic activity by producing regulators of vascular function, including nitric oxide [1], prostacyclin [2], endothelin [3], urokinase- and tissue-type plasminogen activators [4], and plasminogen activator inhibitor-1 [5]. Since the endothelial monolayer functions as a barrier between the blood and subendothelial matrix, damage to vascular endothelial cells can initiate vascular lesions, such as atherosclerosis [6]. When the monolayer is severely or repeatedly injured, platelets aggregate at the damaged site and release transforming growth factor (TGF)-β_1_ from their α-granules in large quantities. Macrophages, T lymphocytes, and vascular endothelial cells release TGF-β_1_ during atherosclerosis progression [7]. Therefore, vascular endothelial cells are largely exposed to TGF-β_1_, and damaged endothelial monolayers are repaired. If cells are exposed to toxic substances, such as heavy metals, during this repair process, TGF-β_1_ may affect their toxicity by regulating the expression of proteins that can modify their toxicities. Elucidating these mechanisms will contribute to the understanding of the interrelationship between toxicity and physiological regulation in vascular endothelial cells.

TGF-β_1_ suppresses cell proliferation [8], reduces fibrinolytic activity by inducing the production of plasminogen activator inhibitor-1 [9], and enhances extracellular matrix components, such as proteoglycans [10,11]. These findings suggest that TGF-β_1_ promotes the repair of damaged blood vessels by maintaining hemostasis and stimulating extracellular matrix formation [12]. The regulation of these endothelial functions by TGF-β_1_ is mediated by the following two receptors: activin-like kinase (ALK)-1, which is selectively expressed in endothelial cells, and ALK5, which is ubiquitously expressed [13]. ALK1 activates the Smad-1, 5, and 8 signaling molecules, whereas ALK5 activates both Smad2/3 and mitogen-activated protein kinases (MAPKs), including extracellular signal-regulated kinase (ERK), p38 MAPK, and c-jun N-terminal kinase (JNK) [14,15,16]. Although the physiological role of TGF-β_1_ has been widely studied, the toxicological significance of TGF-β_1_ exposure in vascular endothelial cells remains poorly understood.

Cadmium is a well-known toxic heavy metal and a risk factor for the onset and progression of atherosclerosis [17,18]. We have previously studied cadmium toxicity on vascular endothelial cells and demonstrated that cadmium injures a monolayer of cells [19], reduces fibrinolytic activity by inducing plasminogen activator inhibitor-1 [20,21], and promotes the synthesis of perlecan, a large heparan sulfate proteoglycan [22]. Heparan sulfate chains also potentiate cadmium cytotoxicity in cells [23]. These results indicate that cadmium toxicity on vascular endothelial cells causes abnormalities in the regulation of cellular functions. In other words, cytokines or growth factors that regulate vascular endothelial cell functions may modify cadmium toxicity in cells. We hypothesized that TGF-β_1_ may be one such cytokine because it regulates various vascular endothelial cell functions, as stated above. In addition, cadmium may further accelerate the progression of vascular lesions by damaging endothelial cells, which are dysfunctional in atherosclerotic vessels.

The intracellular accumulation of cadmium correlates with cadmium cytotoxicity in vascular endothelial cells. Zrt- and Irt-related protein 8 (ZIP8) is a metal transporter that transports cadmium from the extracellular space into the cytosol [24]. The expression of ZIP8 in the vascular endothelial cells of the testis in mouse strains that exhibit a higher sensitivity towards cadmium toxicity was higher than that in the resistant mouse strains [25]. We have previously shown that cadmium induces ZIP8 via cooperative signaling between NF-κB and JNK in vascular endothelial cells [26]. The ZIP transporter expression is regulated by cytokines, such as tumor necrosis factor-α and interleukin-6 [27]. We hypothesized that TGF-β_1_ regulates the expression of endothelial ZIP8 and modifies cadmium cytotoxicity in vascular endothelial cells. In the present study, we report that TGF-β_1_ potentiates cadmium cytotoxicity in vascular endothelial cells by inducing ZIP8. The intracellular signaling pathways involved in ZIP8 induction were also determined.

## 2. Results

### 2.1. Influence of TGF-β_1_ on Cadmium Cytotoxicity towards Vascular Endothelial Cells

**Figure 1:**
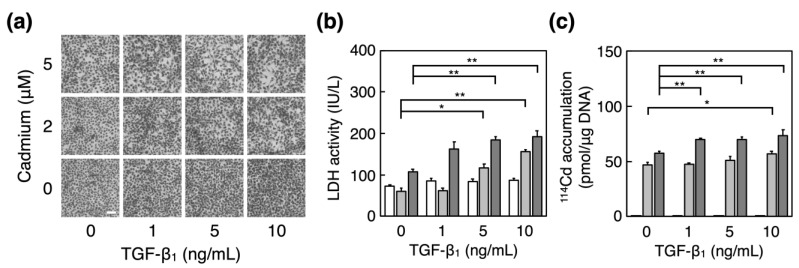
Influence of TGF-β_1_ on cadmium-induced cytotoxicity of vascular endothelial cells. Bovine aortic endothelial cells that were treated with or without TGF-β_1_ (1, 5, or 10 ng/mL) for 24 h were incubated in the presence or absence (white bars) of cadmium at 2 μM (thin gray bars) or 5 μM (dark gray bars) for 24 h. (**a**) This panel shows the cell layer stained with Giemsa. Original magnification (×40). Scale bar = 100 μm. (**b**) This panel shows the conditioned media used to determine lactate dehydrogenase activity, and (**c**) the intracellular accumulation of cadmium, which was measured using inductively coupled plasma-mass spectrometry. Each value represents the mean ± standard error (S.E.) of four independent experiments; and statistical significance compared to the corresponding cadmium-exposed cells without TGF-β_1_ was set as * *p* < 0.05, ** *p* < 0.01.

First, we investigated the effect of TGF-β_1_ pretreatment on cadmium cytotoxicity in vascular endothelial cells using morphological observations and a lactate dehydrogenase leakage (LDH) assay. Morphologically, TGF-β_1_ enhanced cadmium cytotoxicity in vascular endothelial cells (Figure 1a). Specifically, TGF-β_1_ treatment resulted in a larger cadmium-induced de-endothelialized area. The cadmium-induced increase in LDH leakage was significantly increased by TGF-β_1_ treatment (Figure 1b). TGF-β_1_ also increased the intracellular accumulation of cadmium in vascular endothelial cells after exposure to cadmium (Figure 1c), which suggests that TGF-β_1_ potentiated cadmium cytotoxicity in vascular endothelial cells through increased intracellular cadmium accumulation.

### 2.2. TGF-β_1_ Specifically Induces ZIP8 Expression in Vascular Endothelial Cells

**Figure 2:**
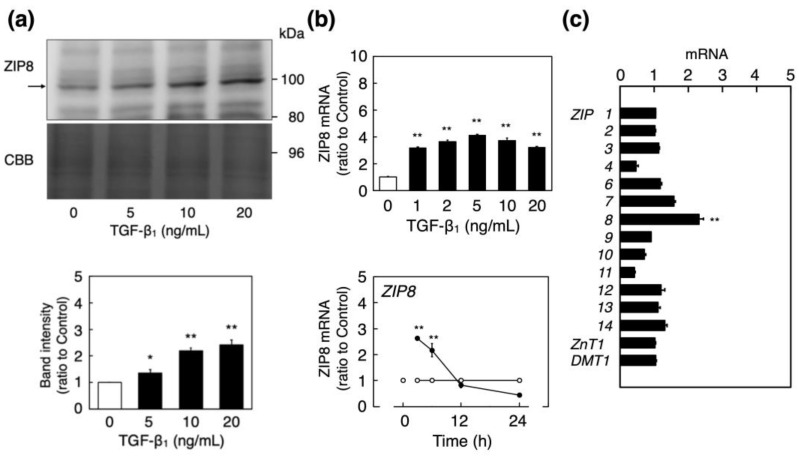
ZIP8 induction in vascular endothelial cells following treatment with TGF-β_1_. (**a**) Bovine aortic endothelial cells were treated with or without TGF-β_1_ (5, 10, or 20 ng/mL) for 24 h, and the expression of ZIP8 protein in the membrane fraction was determined using Western blotting. The band intensity of ZIP8 was normalized by coomassie brilliant blue (CBB) staining, and each value represents the mean ± S.E. of three independent samples; * *p* < 0.05, ** *p* < 0.01 compared with each control. (**b**) Bovine aortic endothelial cells were treated with or without TGF-β_1_ (1, 2, 5, 10 or 20 ng/mL) for 6 h (upper panel) and with (black circles) or without (white circles) TGF-β_1_ (10 ng/mL each) for 3, 6, 12 or 24 h (lower panel). ZIP8 mRNA levels were determined using real-time reverse transcription polymerase chain reaction (RT-PCR). Each value represents the mean ± S.E. of three technical replicates and ** *p* < 0.01 compared with the corresponding control. (**c**) Bovine aortic endothelial cells were treated with or without TGF-β_1_ (10 ng/mL) for 6 h, and ZIP1-4, ZIP6, ZIP7-14, ZnT1, and DMT1 mRNA expression levels were determined by real-time RT-PCR. Each value represents the mean ± S.E. of three technical replicates; ** *p* < 0.01 compared with the control.

Next, we investigated the induction of ZIP8 expression by TGF-β_1_ in vascular endothelial cells. The expression of ZIP8 increased in vascular endothelial cells after treatment with TGF-β_1_, in a concentration-dependent manner (Figure 2a), and TGF-β_1_ increased the expression of ZIP8 mRNA at concentrations of 1 ng/mL and higher (Figure 2b, upper panel). ZIP8 mRNA expression increased 3 and 6 h after treatment with TGF-β_1_, and then decreased to levels below the normal baseline at 24 h (Figure 2b, lower panel). This finding indicated that the transient elevation of ZIP8 mRNA levels that occurred early during the treatment with TGF-β_1_ resulted in ZIP protein induction in vascular endothelial cells. Although TGF-β_1_ significantly increased the expression of ZIP8, that of the other ZIP transporters, ZnT1, and DMT1 did not increase (Figure 2c). This finding suggests that TGF-β_1_ specifically induced ZIP8 expression in these cells.

### 2.3. TGF-β_1_ Increases RSS Levels in Vascular Endothelial Cells

**Figure 3:**
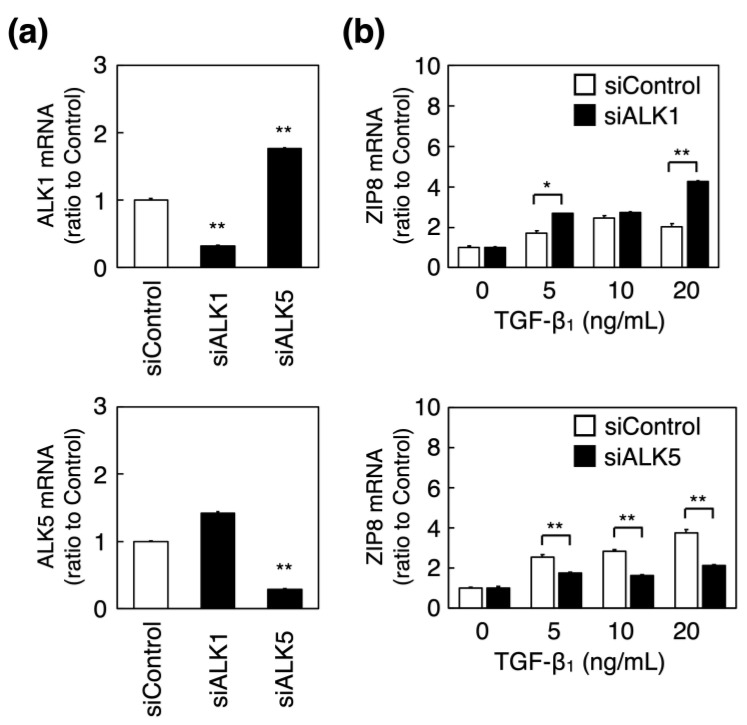
Involvement of ALKs in the induction of ZIP8 by TGF-β_1_ in vascular endothelial cells. (**a**) Bovine aortic endothelial cells were transfected with control, ALK1, or ALK5 siRNAs for 24 h, and ALK1 and ALK5 mRNA expression levels were determined using real-time reverse transcription polymerase chain reaction (RT-PCR). Each value represents the mean ± S.E. of three technical replicates; and ** *p* < 0.01 represents statistical significance compared with the siControl. (**b**) Bovine aortic endothelial cells transfected with the control, ALK1, or ALK5 siRNA for 24 h were treated with or without TGF-β_1_ (5, 10, or 20 ng/mL) for 6 h, and ZIP8 mRNA expression was measured using real-time RT-PCR. Each value represents the mean ± S.E. of three technical replicates; * *p* < 0.05, ** *p* < 0.01 compared with the corresponding TGF-β_1_-treated cells transfected with siControl.

The mechanisms underlying the induction of ZIP8 expression by TGF-β_1_ in vascular endothelial cells were investigated. When ALK1, a receptor for TGF-β_1_, was knocked down in vascular endothelial cells (Figure 3a, upper panel), the levels of ALK5 and TGF-β_1_-induced ZIP8 mRNA increased (Figure 3b, upper panel). In contrast, the TGF-β_1_-induced increase in ZIP8 mRNA levels was significantly suppressed by the knockdown of ALK5, another receptor for TGF-β_1_ (Figure 3b, lower panel). This finding suggests that the induction of ZIP8 expression by TGF-β_1_ is mediated by ALK5, but not ALK1, in vascular endothelial cells.

**Figure 4:**
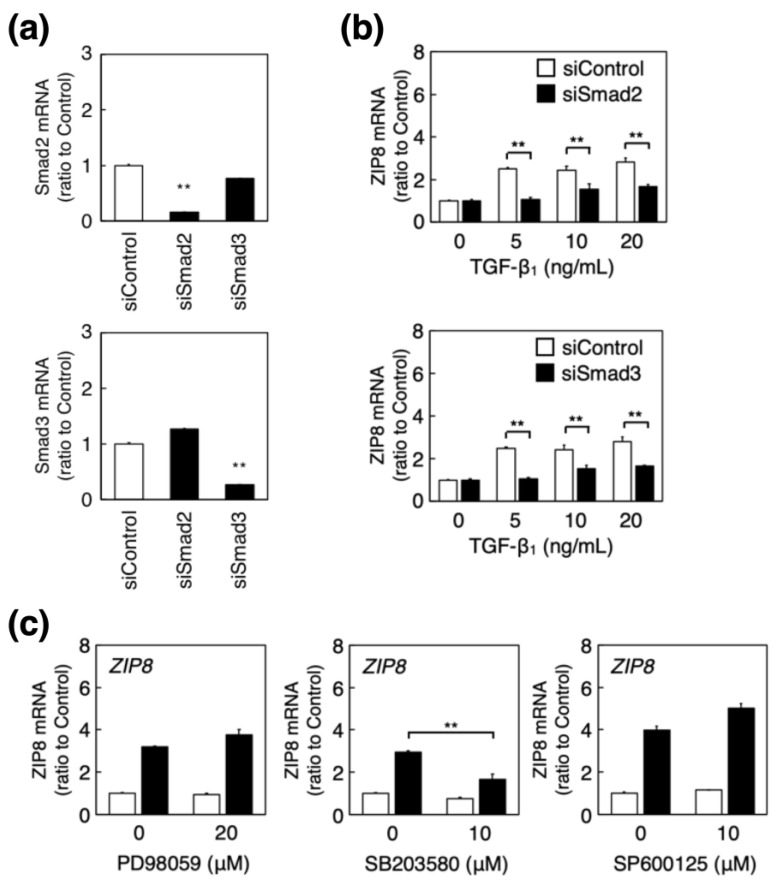
Involvement of Smad2/3 and MAPK signaling in the induction of ZIP8 by TGF-β_1_ in vascular endothelial cells. (**a**) Bovine aortic endothelial cells were transfected with control, Smad2, or Smad3 siRNAs for 24 h, and Smad2 and Smad3 mRNA expression levels were determined using real-time reverse transcription polymerase chain reaction (RT-PCR). Each value represents the mean ± S.E. of three technical replicates; ** *p* < 0.01 represents statistical significance compared with the siControl. (**b**) Bovine aortic endothelial cells transfected with control, Smad2, or Smad3 siRNAs for 24 h were treated with or without TGF-β_1_ (5, 10, or 20 ng/mL) for 6 h, and ZIP8 mRNA expression was determined using real-time RT-PCR. Each value represents the mean ± S.E. of three technical replicates; ** *p* < 0.01 compared with the corresponding siControl. (**c**) Bovine aortic endothelial cells treated with or without PD98059 (20 μM), SB203580, or SP600125 (10 μM each) for 3 h were treated with (black bars) or without (white bars) TGF-β_1_ at 5 ng/mL for 6 h, and ZIP8 mRNA expression levels were determined using real-time RT-PCR. Each value represents the mean ± S.E. of three technical replicates; ** *p* < 0.01 compared with the TGF-β_1_-treated cells without each inhibitor.

ALK5 activates downstream signaling molecules, such as Smad2/3 and MAPKs (ERK, p38 MAPK, and JNK). Our previous study showed that TGF-β_1_ activates Smad2/3, p38 MAPK, and JNK signaling [11] in vascular endothelial cells. Smad2 knockdown (Figure 4a, upper panel) suppressed the TGF-β_1_-induced increase in cellular ZIP8 mRNA levels (Figure 4b, upper panel). When Smad3 was knocked down (Figure 4a, lower panel), the TGF-β_1_-induced increase in ZIP8 mRNA levels was significantly suppressed (Figure 4b, lower panel). These finding suggests that the induction of ZIP8 by TGF-β_1_ was mediated by Smad2 and Smad3. In contrast, pretreatment with SB203580, an inhibitor of p38 MAPK signaling, significantly reduced the TGF-β_1_-induced increase in the ZIP8 mRNA level (Figure 4c, middle panel); however, pretreatment with PD98059 or SP600125, inhibitors of ERK and JNK signaling, respectively, failed to influence the TGF-β_1_ effect (Figure 4c, left and right panels). Altogether, our results suggest that p38 MAPK signaling is involved in the induction of endothelial ZIP8 by TGF-β_1_.

### 2.4. Interaction between TGF-β_1_-Activated Smad2/3 and p38 MAPK Signaling in Vascular Endothelial Cells

**Figure 5:**
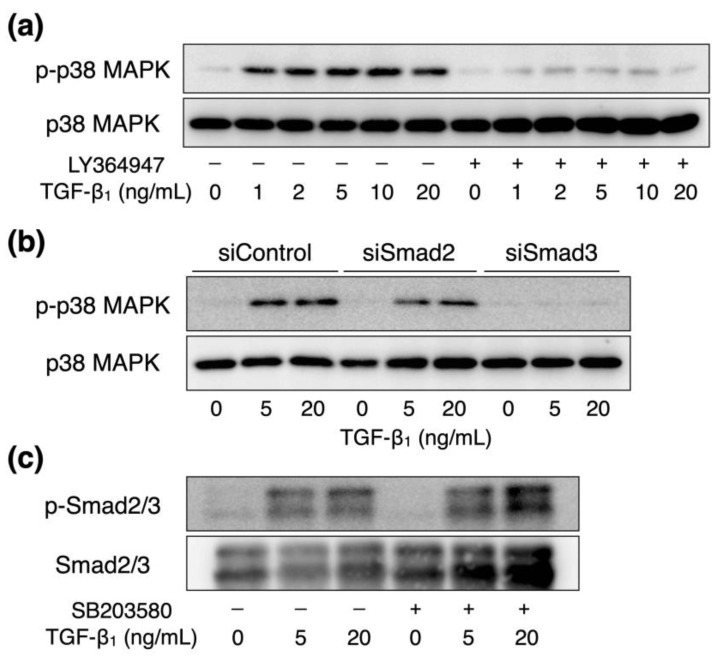
Interaction between TGF-β_1_-activated Smad2/3 and p38 MAPK signaling in vascular endothelial cells. (**a**) Bovine aortic endothelial cells treated with or without LY364947 (5 μM) for 24 h were treated with or without TGF-β_1_ (1, 2, 5, 10, or 20 ng/mL) for 6 h, and the expression levels of phosphorylated p38 MAPK (p-p38 MAPK) and total p38 MAPK proteins were determined using Western blotting. (**b**) Bovine aortic endothelial cells transfected with control, Smad2, or Smad3 siRNAs for 24 h were treated with or without TGF-β_1_ (5 or 20 ng/mL) for 6 h, and the expression levels of phosphorylated p38 MAPK (p-p38 MAPK) and total p38 MAPK proteins were determined using Western blotting. (**c**) Bovine aortic endothelial cells treated with or without SB203580 (10 μM) for 3 h that were treated with or without TGF-β_1_ (5 or 20 ng/mL) for 1 h, and the expression levels of phosphorylated Smad2/3 (p-Smad2/3) and total Smad2/3 proteins were determined using Western blotting.

The involvement of ALK5-Smad2/3 signaling in the activation of p38 MAPK by TGF-β_1_ in vascular endothelial cells was next investigated. We previously revealed that the activation of p38 MAPK signaling by TGF-β_1_ is regulated by Smad3 [11]. TGF-β_1_-induced phosphorylation of p38 MAPK protein was reduced following pretreatment with LY364947, an inhibitor of ALK5 (Figure 5a). Smad3 knockdown suppressed TGF-β_1_-induced phosphorylated p38 MAPK protein expression; however, Smad2 knockdown failed to suppress this effect (Figure 5b). In contrast, pretreatment with SB203580, an inhibitor of p38 MAPK, did not affect the phosphorylation of Smad2 and Smad3 proteins (Figure 5c). Our findings suggest that the activation of p38 MAPK signaling by TGF-β_1_ was mediated by ALK5-Smad3 signaling under these experimental conditions.

## 3. Discussion

Since TGF-β_1_ is released from α-granules of platelets that aggregate at the damaged vascular endothelial monolayers, modification of cadmium cytotoxicity against vascular endothelial cells by TGF-β_1_ appears important to facilitate understanding of the damage to the cells caused by cadmium while the damaged endothelium is being repaired. The present study was conducted to confirm the hypothesis that cadmium cytotoxicity is mediated by TGF-β_1_. The following results were obtained: (1) TGF-β_1_ potentiated cadmium cytotoxicity in vascular endothelial cells; (2) TGF-β_1_ increased the intracellular accumulation of cadmium in cells; (3) TGF-β_1_ induces ZIP8 expression; (4) this induction was mediated by the ALK5-Smad2/3 signaling pathway, which can be partially influenced by p38 MAPK. These results suggest that TGF-β_1_ potentiated cadmium cytotoxicity by increasing the intracellular accumulation of cadmium, which was caused by the induction of ZIP8 via the ALK5-Smad2/3 and ALK5-Smad3-p38 MAPK signaling pathways in vascular endothelial cells. Since ALK5 is a decoy receptor of ALK1, it is possible that TGF-β_1_ binds to ALK5 more readily and promotes the activation of ALK5-downstream signaling by knockdown of ALK1. Cadmium may exhibit stronger cytotoxicity to vascular endothelial cells during the damaged endothelium repair process.

Free zinc ions function as second messengers, and their intracellular concentration is strictly regulated by ZIP, ZnT transporters, and zinc-binding proteins, such as metallothionein [28,29]. Because ZIP8 transports zinc ions into cells, the induction of ZIP8 by TGF-β_1_ can contribute to a transient increase in free intracellular zinc ions. In contrast, zinc ions suppress the activation of Smad3 and NF-κB [30,31], which suggests that excess activation of TGF-β_1_ signaling, including Smad3 and NF-κB activation, is suppressed by an increase in zinc ions via the induction of ZIP8 in vascular endothelial cells after exposure to TGF-β_1_. In other words, the induction of ZIP8 by TGF-β_1_ can function as a negative TGF-β_1_ signaling feedback mechanism. The modulation of TGF-β_1_ activity by ZIP8 may play a role in the prevention of vascular disorders, since an epidemiological study showed that genetic polymorphisms of ZIP8 correlated with increased cardiovascular disease mortality [32]. The present data suggest that cadmium exploits the biological system that modulates TGF-β_1_ activity by inducing ZIP8 expression to intensify vascular endothelial cell cytotoxicity.

Smad2 and Smad3 serve as both transcription factors and signaling molecules. Specifically, Smad2/3 binds to Smad-binding elements (SBEs) on the promoter region of target genes and functions as a transcriptional factor [33]. Smad3 has an MH1 domain that recognizes and binds to SBEs. We postulate that Smad2 is directly binds to the ZIP8 promoter region as a transcription factor and induces ZIP8 expression in vascular endothelial cells. The induction may have been mediated by the downstream transcription factor(s) of Smad2, as a signaling molecule, although we have not yet identified the factor(s). In contrast, Smad3 is involved in p38 MAPK activation, as demonstrated in our previous study [11]. Smad3, as a signaling molecule, may induce endothelial ZIP8 expression via activation of p38 MAPK signaling. However, Smad3 does not have a specific amino acid sequence near the MH1 domain, unlike Smad2. Therefore, the ability of Smad3 to bind to SBEs is stronger than that of Smad2 [34]. It is possible that Smad3 and Smad2 directly bind to the MH1 domain and induce ZIP8 expression in vascular endothelial cells. Previously, we reported that TGF-β_1_ modulates the expression of syndecan-4, a transmembrane small heparan sulfate proteoglycan, in a biphasic manner [11]. Furthermore, the Smad3-p38 MAPK pathway mediates the early upregulation of syndecan-4 by TGF-β_1_, whereas late downregulation is mediated by the Smad2/3 pathway. Unlike this modulation of syndecan-4 expression, both Smad2/3 and Smad3-p38 MAPK pathways have been suggested to mediate the upregulation of endothelial ZIP8 expression by TGF-β_1_. However, a transient increase and subsequent suppression of endothelial ZIP8 levels after TGF-β_1_ treatment were observed in the present study. Therefore, biphasic regulation may be characteristic of TGF-β_1_-ALK5-Smad2/3 signaling.

The results of the present study revealed that TGF-β_1_ potentiates cadmium cytotoxicity in vascular endothelial cells by inducing ZIP8 expression via the ALK5-Smad2/3 signaling pathway. TGF-β_1_ may be one of the factors that determine the strength of cadmium toxicity in target organs. Cytokines other than TGF-β_1_, including tumor necrosis factor-α [35] and interleukin-6 [36], may modify the toxicity of cadmium in vascular endothelial cells and other cell types. Further studies on regulating endothelial ZIP8 expression and modifying cadmium cytotoxicity by regulators are required to attain an improved understanding of cadmium toxicity in target organs and blood vessels.

## 4. Materials and Methods

### 4.1. Materials

Bovine aortic endothelial cells were purchased from Cell Applications (San Diego, CA, USA). Dulbecco’s modified Eagle’s medium (DMEM) and calcium- and magnesium-free phosphate-buffered saline (CMF-PBS) were purchased from Nissui Pharmaceutical (Tokyo, Japan). Tissue culture plates and dishes were purchased from AGC Techno Glass (Shizuoka, Japan). Fetal bovine serum (FBS), OPTI-MEM reduced serum medium, Lipofectamine RNAiMAX transfection reagent, and a high-capacity complementary DNA (cDNA) reverse transcription kit were purchased from Thermo Fisher Scientific (Waltham, MA, USA). May-Grünwald and Giemsa staining solutions were purchased from Merck KGaA (Darmstadt, Germany). CytoTox 96 Non-Radioactive Cytotoxicity Assay (a LDH kit) was purchased from Promega (Madison, WI, USA). The rabbit polyclonal anti-ZIP8 antibody (A10395) was purchased from ABclonal (Tokyo, Japan). QIAzol Lysis Reagent was purchased from QIAGEN (Venlo, Netherlands), and GeneAce SYBR qPCR Mixα was purchased from Nippon Gene (Tokyo, Japan). The TGF-β_1_ and ALK5 inhibitor LY364947 were purchased from FUJIFILM Wako Pure Chemical Corporation (Osaka, Japan). ERK (PD98059), p38 MAPK (SB203580), and JNK (SP600125) inhibitors were purchased from Cayman Chemical (Ann Arbor, MI, USA). Rabbit polyclonal anti-phospho-p38 MAPK (Thr180/Tyr182) (#9211), rabbit polyclonal anti-p38 MAPK (#9212), rabbit monoclonal anti-phospho-Smad2/3 (Ser423/Ser425) (#8828), rabbit monoclonal anti-Smad2/3 (#8685), and horseradish peroxidase-conjugated anti-rabbit IgG (#7074) were purchased from Cell Signaling Technology (Danvers, MA, USA). Chemi-Lumi One L and the other reagents were purchased from Nacalai Tesque (Kyoto, Japan).

### 4.2. Cell Culture and Treatment

Vascular endothelial cells were cultured at 37 °C in 5% carbon dioxide in DMEM supplemented with 10% FBS until they reached confluency. The medium was removed, and the cells were washed twice with serum-free DMEM. The cells were then incubated in the presence or absence of cadmium (2 or 5 μM) or TGF-β_1_ (1, 2, 5, 10, or 20 μM) for 3, 6, 12, or 24 h in serum-free DMEM in 100 mm dishes in 6- or 24-well culture plates. The blood concentration of cadmium in humans has been reported to be less than 1 µM [37,38,39]. Using these data as a reference, we set the concentration to 2 µM and 5 µM, to evaluate the cytotoxicity of cadmium over a short interval using a culture system.

### 4.3. Small Interfering RNA Transfection

Transfection of small interfering RNAs (siRNAs) (Nippon Gene, Tokyo, Japan) was performed as previously described [26]. Briefly, the annealed siRNA duplex and Lipofectamine RNAiMAX were dissolved in Opti-MEM in separate tubes and incubated for 5 min at room temperature, followed by mixing and incubation for 20 min at room temperature. Vascular endothelial cells were grown to confluence in DMEM supplemented with 10% FBS, and then incubated for 4 h in fresh DMEM supplemented with 10% FBS and the siRNA/Lipofectamine RNAiMAX mixture. The final concentrations of siRNA and Lipofectamine RNAiMAX were 40 nM and 0.2%, respectively. After 24 h, cells were incubated in the presence or absence of cadmium chloride or TGF-β_1_. The sequences of the sense and antisense strands of the siRNAs were as follows: bovine ALK1 siRNA, 5-UUCAUGUCCUCAAAGCUGGGG-3 (sense) and 5-CCAGCUUUGAGGACAUGAATT-3 (antisense); bovine ALK5 siRNA, 5-UUCAUUUGGCACUCGAUGGUG-3 (sense) and 5-CCAUCGAGUGCCAAAUGAATT-3 (antisense); bovine Smad2 siRNA, 5-UUCAAAACCCUGAUUAACGTT-3 (sense) and 5-CGUUAAUCAGGGUUUUGAATT-3 (antisense); bovine Smad3 siRNA, 5-UGUUUUCGGGGAUGGAAUGTT-3 (sense) and 5-CAUUCCAUCCCCGAAAACATT-3 (antisense). A nonspecific sequence was used as the siRNA negative control (Qiagen).

### 4.4. Morphological Appearance and Lactate Dehydrogenase Assay

Vascular endothelial cells cultured in 24-well culture plates were incubated in the presence or absence of cadmium for 24 h after pretreatment with TGF-β_1_ for 24 h. The conditioned medium was harvested after incubation, and aliquots were used to measure LDH activity. The absorbance of each sample was measured at 490 nm wavelength using a Multiskan FC microplate reader (Thermo Fisher Scientific). The cell layers were washed with CMF-PBS, fixed with methanol, and stained with Giemsa stain for morphological observation.

### 4.5. Intracellular Accumulation of Cadmium

Vascular endothelial cells cultured in 6-well culture plates were incubated in the presence or absence of cadmium for 24 h after pretreatment with TGF-β_1_ for 24 h. The conditioned medium was discarded after incubation, and the cells were washed twice with ice-cold CMF-PBS and harvested in 1 mL of CMF-PBS. The cell suspensions were sonicated to prepare the homogenates; 0.6 mL of the homogenates were added in 4.4 mL of 0.1 M nitric acid and used to detect intracellular cadmium (*m*/*z* = 114) via inductively coupled plasma mass spectrometry (NexION 300S; PerkinElmer, Waltham, MA, USA). Another portion of the lysate was analyzed for DNA content using the fluorometric method [40], and the cadmium content was expressed as pmol/μg DNA.

### 4.6. Extraction of the Membrane Fraction

The membrane fraction was extracted according to a previously described method [26]. Briefly, vascular endothelial cells cultured in 100 mm culture dishes were exposed to TGF-β_1_ (5, 10, or 20 ng/mL) for 24 h, and the cell layer was washed twice with CMF-PBS and harvested in 1.3 mL of ice-cold CMF-PBS. The cell suspensions were centrifuged at 12,000× *g* for 3 min, the supernatant was discarded, and the pellets were suspended in 20 mM 4-(2-hydroxyethyl)-1-piperazineethanesulfonic acid (HEPES) solution containing 250 mM sucrose and 1 mM ethylenediaminetetraacetic acid (EDTA) and sonicated for three cycles (5 s pulse and 5 s rest, on ice) using an Ultrasonic Homogenizer UR-20P (TOMY SEIKO, Tokyo, Japan). The homogenates were centrifuged at 5000× *g* for 5 min, and the supernatants were transferred into new tubes and centrifuged at 15,000× *g* for 30 min. The supernatants were discarded, and the pellets were lysed in 50 mM HEPES solution (pH 7.5) containing 150 mM sodium chloride, 0.5% 3-[(3-cholamidopropyl)-dimethylammonium]-1-propanesulfonate, 0.1 mM ethylene glycol tetra-acetic acid, and 0.1 mM EDTA, and analyzed using Western blotting as described below.

### 4.7. Western Blot Analysis

Vascular endothelial cells in 6-well culture plates were exposed to cadmium or TGF-β_1_ for 6 or 24 h. The conditioned medium was then discarded, and the cells were lysed in a sodium dodecyl sulfate sample buffer (50 mM Tris-HCl buffer solution containing 2% sodium dodecyl sulfate and 10% glycerol, pH 6.8) and incubated at 95 °C for 5 min. Next, 2-mercaptoethanol and bromophenol blue (1.67% each) were added to the samples (10 µg protein) and incubated at 95 °C for 3 min. Cellular proteins were separated via sodium dodecyl sulfate-polyacrylamide gel electrophoresis on 8% or 12% polyacrylamide gels and electrotransferred to polyvinylidene difluoride membranes at 2 mA/cm^2^ for 1 h. The membranes were blocked with 2% bovine serum albumin in 20 mM Tris-HCl buffer solution containing 15 mM NaCl and 0.1% Tween 20 (pH 7.5), and then incubated with primary antibodies (1:1000). After washing with a 20 mM Tris-HCl buffer solution containing 15 mM NaCl and 0.1% Tween 20 (pH 7.5), the membranes were incubated with horseradish peroxidase-conjugated secondary antibodies (1:5000) for 1 h. Immunoreactive bands were visualized via enhanced chemiluminescence using Chemi-Lumi One L and scanned with an LAS-3000 (Fujifilm, Tokyo, Japan). The ZIP8 band intensity was normalized using Coomassie Brilliant Blue staining.

### 4.8. Real-Time Reverse Transcription Polymerase Chain Reaction

Vascular endothelial cells in 6-well culture plates were incubated in the presence or absence of TGF-β_1_ for 3, 6, 12, or 24 h after pretreatment with PD98059, SB203580, or SP600125 for 3 h. After incubation, the conditioned medium was discarded, and the cell layer was lysed with 500 μL of QIAzol Lysis Reagent. Chloroform (125 μL) was mixed with the lysate, and the mixture was centrifuged at 15,000× *g* for 15 min. The supernatant was harvested, mixed with an equal volume of 70% ethanol, and centrifuged at 15,000× *g* for 10 min, after which the supernatant was discarded. The precipitate was resuspended in 70% ethanol, centrifuged at 12,000× *g* for 5 min, and the precipitate containing the total RNA was dried. cDNA was synthesized using a high-capacity cDNA reverse transcription kit. Real-time polymerase chain reaction was performed using GeneAce SYBR qPCR Mix α with 10 ng cDNA and primers on a StepOnePlus Real-Time PCR system (Thermo Fisher Scientific). The thermal cycling parameters were as follows: 95 °C for 10 min, followed by 45 cycles of 95 °C for 30 s, and 60 °C for 1 min. The mRNA levels of ZIP1-4, ZIP6, ZIP7-14, ZnT1, divalent metal transporter 1 (DMT1), ALK1, ALK5, Smad2, Smad3, and glyceraldehyde 3-phosphate dehydrogenase (GAPDH) mRNA were quantified using the comparative Ct method, and the fold change in the intensity value of the target gene was normalized to that of GAPDH. Sequences of the forward and reverse primer strands are listed in Table 1.

### 4.9. Statistical Analysis

The data were analyzed for statistical significance using analysis of variance, Bonferroni’s multiple *t*-test, or Student’s *t*-test, when possible. Statistical significance was set at *p* < 0.05.

## Figures and Tables

**Table 1:** Primers for real-time RT-PCR used in this study.

Gene	Forward Primer (5′→3′)	Reverse Primer (3′→5′)
ZIP1	TTCTCTACATCACCTTCCTGG	AACCTTCCTTGCCTGTCTTG
ZIP2	GCTCTCGCTCTCCTTTCAC	ACCAGCCGCAGTCCTACA
ZIP3	GGACACACTCACCTCAACGC	CTCAAGGCTCCAAGCAGAAC
ZIP4	GACAGCCACAGTGACGACAG	CAGACATTCCGTACACAGCC
ZIP6	CCTGAAAATGATGATGATGTGG	CAAGATTGCTGGCTGCTGAG
ZIP7	TATTCTATGTAGCAACGGTGTC	CGAGGTGGCAATCAAC
ZIP8	GAATGAGCACTCGACAAGCC	TAGAGGAACATGCCTCCAGC
ZIP9	CAGCCTCTTGTCTCGCCTTG	ATGTCTGTATCCTTCGCAGTGTG
ZIP10	TTCTATCACTGTCATTAGCCTGC	GCGTCTCCACTCATTGTTCC
ZIP11	CCATCACCATCCACAACATC	TACCAGAAGGCTCTCCAGG
ZIP12	GAGGACAGATGGAAGGCAAG	TGATGTAATAGAGGAGGAGAAGAG
ZIP13	CTGGACAGTAAGGAGAGCGAG	GAGCAGGTGGAAGAAACACG
ZIP14	TCTCGGTAGTGCCTCTGTCC	GAATGTCTCAGTGCTGGTTGG
ZnT1	CAACAGCAGCAACTCCAACG	CCAGTCTTATCTTCATCCTCTTCC
DMT1	CACAGGTAGCCATCAGAGCC	ACCAGGTTAGGAGTTCAGGAG
ALK1	CAACCACTACTGCTGCTACA	CCATCTCCTTGAGGCTGC
ALK5	GTCTGCTTTGTCTGTATCTCACTCA	TCCTCTTCATTTGGCACTCG
Smad2	CAGAATACCGAAGGCAGACG	TGAGCAACGCACTGAAGG
Smad3	ACTACAGCCATTCCATCC	ATCTGGTGGTCACTGGTCTC
GAPDH	CAATGACCCCTTCATTGACCTTC	GGATCTCGCTCCTGGAAGATG

## Data Availability

Not applicable.
